# The Cause of Caries—Bacteria or Acids?

**Published:** 1883-03

**Authors:** F. Y. Clark


ARTICLE II.

### THE CAUSE OF CARIES—BACTERIA OR ACIDS?

[Read before the Brooklyn Dental Society, Jan. 8, 1883.]

BY F. Y. CLARK, M.D., D.D.S.

*Mr. President:* Through your complimentary invitation I am here to-night to reopen the subject of your last meeting, which was, as you know, "Bacteria of the Teeth—Acids or Germs, which?"

In order to bring the subject more properly before you, I will attempt a brief description of bacteria in general, and then refer more particularly to those found in the mouth and about the teeth, which are claimed to be the real cause of caries.

The organisms called bacteria have been known for over a century; but their nature and connection with disease, until recently, has been little understood. They were for a long time classed with infusoria and other low forms of animal life, but now, thanks to microscopical advancement, are better known, and acknowledged to be of vegetable origin, although when we view their actions—turning, twisting and gliding, with as much or more activity than the lower animal organisms—it is difficult to understand how they can be so classed. With these infinitesimal forms it would seem as though the vegetable and animal ran into each other and became united, and were it not for their sustenance by absorption and their mode of propagation there would be little or no ground for separating them from infusoria or low forms of animal life. They are the "first, smallest, and simplest forms of life, and beyond them, so far as microscopical evidence goes, life does not exist." "Could we," as Cohn says, "view a man under the power used for bringing to view some of these forms, he would appear as large as Mount Blanc, or even Chimborazo; but even under this colossal amplification, the smallest bacteria do not appear larger than the points of commas of good print. In microscopically comparing the diameters of some of these organisms found in the mouth with the bioplastic mesh tubes of the dentine, it is found they have ample room to form a colony in a single mesh. Under favorable circumstances, with moist air, warm temperature, and suitable pabulum, their increase, which is by fissation or bi-partition, is beyond conception. Within a few days one bacterium will multiply so that figures almost fail to express them. Thus, what they lack in size they make up in numbers. When a swarm is viewed under the microscope and a request made to count them, one glance is sufficient to deter any one from such a task. As no doubt most of you know, bacteria are found in all impure matters and fluids in damp places, and in all matters, vegetable and animal, undergoing decomposition. As is seen in fermentation, their rule is to decompose and reproduce. It is only recently that American scientists have given this field of discovery much attention. Nearly all we know of the life history of these forms has been gained from French or German investigators. But the names and classification of the most of these writers are contradictory, inapplicable, and to the American tongue, almost unpronouncable. Dr. Ferdinand Cohn's description and classification is by far the best—yet with all his apparent accuracy, it requires long and close study to find the organisms as he has placed them. If investigators would give the amplification with these plates and culture fluid, there would be little or no trouble in making out and reproducing the forms as described and plated.

As it is necessary to refer to the species connected with this subject further on, we submit Cohn's classification. He gives six genera, as follows:

1st. Micrococcus—ball or egg-shaped bacteria.
2nd. Bacterium—short, rod-like.
3d. Bacillus—straight, fiber-like.
4th. Vibrio—wavy, curl-like.
5th. Spirillum—short, screw-like.
6th. Spirochate—long, flexible, spiral.

Of the first he gives six species:
" " 2nd " " 4 "
" " 3d " " 2 "
" " 4th " " 2 "
" " 5th " " 3 "
" " 6th " " 1 "

The species found in the mouth and about the teeth are the micrococcus, vibrio regula, spirochati plicatilis, bacteria termo, and leptothrix. Under certain conditions, when the teeth are for some time undisturbed with the brush, as stated in former papers, through advanced fermentation, other species are now and then seen, but those named are the species oftenest met with. At one time, before microscopes were known, spherical coloring bacteria played an important role in the world's history. They were known as wonder blood, and when they fell on a slice of bread or cooked sliced potatoes, and gradually spread over the surface it was considered a bad omen, and frequently priests and witches, preying on the superstition of the ignorant and wealthy, made these blood spots, as they called them, the source of much gain. Cohn, in referring to these spots, says: "It has long been a saying, that suddenly, from time to time, a drop of blood would form on food, and especially on bread, and so increase that it would spread over wide surfaces. This was observed in ancient times, and it was held that it was a sign of threatened disaster, and showed the anger of God, disclosed secret guilt, and called for bloody atonement, and history records numberless sacrifices which, till recent times, were offered by the superstitious as often as this wonder of blood was seen on food, but especially if on the consecrated wafer.

With a century of enlightenment the blood wonder gradually ceased; but only within the last ten years do we know that the wonderful appearance had a scientific origin. It was Ehrenberg who first investigated this appearance of blood most carefully. It formed itself in moist air on cooked, not on raw food; on potatoes, rice, paste, also on flesh, milk and on white of egg, and that of itself without any one voluntarily causing it to be produced. At first it appeared as a very small rosy red or purple slimy drop, which grew to the size of a pin head and appeared like fish spawn; then it flattened, ran together, and formed a thick bloody slime. If one spread out the drop of gelatine-like substance on a fresh potato it would multiply rapidly, and it has with ease been increased to so great a quantity that it could be used for coloring; unfortunately this coloring material is not durable, being soon destroyed by light. Ehrenberg found in it the red, slimy numberless little oval bodies to which he gave the name of *Monas prodigiosus;* we designate them better as red spherical bacteria (*Micrococcus prodigiosus*). They nourish themselves on the albumen contained in the food on the surface of which they develop, decompose the same, and generate by a peculiar pigment fermentation the red coloring matter which possess a striking relation to that briliant aniline color which at present time is of so much value to the dyeing industry.

In historical interest and in the mighty impression which it exercised on the myth-forming fancy of the people, the wonder blood stands alone; as a physical phenomenon it shows a whole series of colors, which appear almost as a rule in moist places, on potatoes, cheese, cooked eggs and other foods; in appearance snow white, sulphur yellow, spanish green, violet blue or brown; all these colors which originate from spherical bacteria which under the microscope can scarcely be distinguished from the *micrococcus prodigiosus* of the wonder blood.

We give little more space to this part of our subject because little has been written on it, and acid advocates argue that the shades met with in carious tooth structure cannot be produced by bacterial organisms, whereas just the reverse is the fact, as all and every shade of caries can be readily accounted for and understood when we study the coloring power of the micrococcus found in carious dentine. By placing a few layers of sound dentine in a few drops of saliva and keeping it at a normal temperature a few days, nearly all the shades met with in caries can be produced.

Prof. Tyndall says, in his paper on floating matter in the air, that fifty per cent. of ordinary floating dust is organic. This being the case it is easy to understand how a speck of living matter can enter the mouth and, finding a home in a pit-fissure, or some unmolested locality, can propagate and, entering the mesh tubules, either directly or by fermentation, bring into existence other species that absorb the bioplasm in the mesh work of the dentine, for all investigation goes to show that these forms live at the surface of the substances on or in which they are found; and as they are found in the tube cells of the dentine in large numbers, and particularly where caries is in rapid progress, it is reasonable to connect them with the cause. Perhaps we can better understand this by filling an ounce bottle with sterilized matter. If this is properly done and hermetically sealed, the matter will remain forever, or as long as the seal is hermetic, without change; but if the seal is punctured with the finest needle or wire, air germs enter and other forms follow within a few hours. In speaking of fermention we do not allude to the ordinary known ferments, for there are changes in all matters undergoing this process before any ferment known sets in. Pasteur, as far as he goes, is perhaps the best authority on fermentation, but he says nothing of the organic change before alcoholic fermentation. It is by no means necessary that mycoderma aceti—the ferment organism of acetic acid—should be seen before bacteria enters the mesh tubes. This is done before alcoholic fermentation, and even before mycoderma is seen; therefore organic action on tooth structure is apparently independent of acetic acid. It may be that in exceptional cases this ferment may help to open the gate through the enamel, but we have never seen a case where in the mouth this acid was of sufficient strength to do it. The experiments of Prof. Mayr and Dr. Spaulding stand as bulwarks of stubborn facts against such a theory, and until they are demolished and washed away there is little or no need of further defence. It is all very well to prate about acetic acid destroying or dissolving the lime salts and prove this by placing a tooth in vinegar; but that is out of the mouth, not in. Where there is a constant flow of saliva this acid has not time to generate sufficient strength to act on tooth structure. About the entrance of a carious cavity, which has been undisturbed for a few days, acid may be found, but never where the real battle is going on—where the dead and living meet at this border line, were it possible to show it, a fight could be seen between two sets of organisms, the invaders and the invaded, the one attempting to close their gateways with ossific plasm and the other by absorbing this plasm before calcification augments its force, and unless prevented by some outer force complete disintegration of the tooth is the result. Perhaps if we view a tooth as an amalgam filling a better understanding of what we mean or wish to convey may be had. The mercury holds the powder of which the filling is composed together, converting all into a hard homogeneous mass; force out the mercury and disintegration follows. Just so with a tooth; remove the mesh that permeates every part of both enamel and dentine and disintegration of tooth structure is the result. This is the *modus operandi* of bacteria. They absorb the bioplasm in the mesh which binds the hard and limy structure, leaving the lime salts disintegrated, just like the amalgam powder.

Now, let us sum up the evidence introduced in favor of bacterian action and see how we stand. Let us see if we have not enough facts to justify a stronger belief in organic caries by bacteria than by acids.

1st. Acids cannot produce the different shades met with in carious tooth structure, while by the coloring powers of bacteria all these shades can be easily accounted for.

2d. If acids in the fluids of the mouth were the cause, the enamel, owing to its limy nature would be dissolved or equally attacked all over its surface, instead of here and there in obscure localities.

3d. In many well-authenticated cases where decay was in rapid progress, when acids were expected none were found.

4th. In many cases where decay is going on, bacteria are found not only in the fluids, but about the teeth and amid the carious structure.

5th. It is a well-established fact that seldom, if ever, does decay take place on a smooth, well-polished surface, either of enamel or dentine.

6th. The mode of attack is not that of an acid. A minute, slightly-colored speck or stain makes its appearance, and, penetrating the enamel, seeks the mesh work of the dentine; acids do not act in this way; the lime in the enamel would be most to their liking.

7th. If acids caused the trouble, the free use of alkaline lotions and powders and the filthy practice recommended by a few dental savants, of packing the teeth over night with chalk, ought to be beneficial if not effectual; but this has been tried, and we believe we express the opinion of the majority in saying it has failed to prove productive of any positive good. If caries were produced by acids, then every tooth before filling should be washed out with an alkali. This would be intelligent and rational practice, but instead of this many advocates of the acid theory use creosote and carbolic acid before filling, and use it just because experience teaches them that it is good.

8th. Observation proves that nearly all the numerous cases of recalcification are produced by some antiseptic, such as mercury, nicotine, or carbolic acid, as every practitioner of any experience has noticed. If we seek a cause for this we are led to but one solution of the problem. Such agents are auxiliaries, *and when brought forward hold the invaders in check while the invaded close the mesh gates with ossific plasm and by this wall of recalcification save the citadel.*

Since this paper was written and read we have seen Dr. W. D. Miller's able and interesting essay in the January number of the "Cosmos." He says bacteria penetrate beyond the line of decay, and that the bacilli and micrococci are found in large numbers far into the dentine; notwithstanding this, he rather leans to the acid theory. These forms being found in the mesh tubes of the dentine, we naturally ask, "How came they there?" "What are they there for?" "Can they live on nothing and increase in numbers?" There is but one way to answer these questions. We know these organisms are never found except where there is a change of matter or substance. They always live and increase at the sacrifice of the substance on or in which they are found. They enter the mesh work of the teeth either by spontaneous generation or through the agency of air-germs. We cannot for one moment believe in their equivocal origin, but believe, as often stated, they originate from air-germs. If this is so, the species plated by Dr. Miller must have developed from air-germs through some kind of fermentation in the pit-fissures or the place of lodgment. But, according to Pasteur, the organisms, as described and plated, are not of a kind to bring on acetic fermentation. We have no doubt as to some kind of fermentation in the outer part of many decayed cavities, but further on, just beyond the line of decay, where the evil is being enacted, nothing is at work but organisms found and described. Therefore we cannot see why Dr. Miller, after proving the presence and action of bacteria, should doubt their work.

---

ARTICLE III.

